# Modelling Impact of High-Rise, High-Density Built Environment on COVID-19 Risks: Empirical Results from a Case Study of Two Chinese Cities

**DOI:** 10.3390/ijerph20021422

**Published:** 2023-01-12

**Authors:** Yong Xu, Chunlan Guo, Jinxin Yang, Zhenjie Yuan, Hung Chak Ho

**Affiliations:** 1 School of Geographical Science and Remote Sensing, Guangzhou University, Guangzhou 510006, China; 2 Department of Geography and Resource Management, The Chinese University of Hong Kong, Hong Kong 999077, China; 3 Department of Anesthesiology, School of Clinical Medicine, The University of Hong Kong, Hong Kong 999077, China

**Keywords:** urban environment, COVID-19, infection rate, population

## Abstract

Characteristics of the urban environment (e.g., building density and road network) can influence the spread and transmission of coronavirus disease 2019 (COVID-19) within cities, especially in high-density high-rise built environments. Therefore, it is necessary to identify the key attributes of high-density high-rise built environments to enhance modelling of the spread of COVID-19. To this end, case studies for testing attributes for modelling development were performed in two densely populated Chinese cities with high-rise, high-density built environments (Hong Kong and Shanghai).The investigated urban environmental features included 2D and 3D urban morphological indices (e.g., sky view factor, floor area ratio, frontal area density, height to width ratio, and building coverage ratio), socioeconomic and demographic attributes (e.g., population), and public service points-of-interest (e.g., bus stations and clinics). The modelling effects of 3D urban morphological features on the infection rate are notable in urban communities. As the spatial scale becomes larger, the modelling effect of 2D built environment factors (e.g., building coverage ratio) on the infection rate becomes more notable. The influence of several key factors (e.g., the building coverage ratio and population density) at different scales can be considered when modelling the infection risk in urban communities. The findings of this study clarify how attributes of built environments can be applied to predict the spread of infectious diseases. This knowledge can be used to develop effective planning strategies to prevent and control epidemics and ensure healthy cities.

## 1. Introduction

The coronavirus disease 2019 (COVID-19) has emerged as an international public health emergency and affected millions of people worldwide since its outbreak in late 2019. According to the World Health Organization, as of 6 January 2023, more than 0.65 billion people have been infected across more than 188 countries and territories, and 6.6 million people have died. Compared with previous outbreaks of severe acute respiratory syndrome and Middle East respiratory syndrome, COVID-19 is more contagious and more widely transmitted within the communities [1]. The high infection rate coupled with large-scale population movement, particularly in large and densely populated cities, have increased the speed and scope of disease spread, and adversely affected public health and safety, and economic development [2].

The outbreak of COVID-19 has stimulated multidisciplinary research pertaining to its origins and mechanisms [3]. Epidemiologists have used mathematical models to understand its development and transmission patterns [4], and public health scientists and geographers have used geographic information system (GIS) data to explore COVID-19 risk factors and spreading patterns [5]. Overall, research on the risk factors and transmission routes has attracted substantial interest across domains.

The spread of COVID-19 is influenced by socioeconomic factors (e.g., population density [6], income, and race [7]), built-environment factors (e.g., road density [8], building density [9], floor area ratio [10], and parks and vegetation [11,12]), environmental factors (e.g., air pollution [13] and humidity and temperature [14]), and public service factors (facilities such as bus stations, clinics, and restaurants [15]). The factors driving the spread of COVID-19 may differ by scale, e.g., country, city, and urban district [16]. At the country or city scale, the infection rate of COVID-19 is mainly influenced by human factors such as population density, per capita GDP, population mobility, and travel intensity [8]. Large and densely populated cities are typically associated with high infection risk. With the increase in the available infection data, several researchers have investigated the impact of urban environments on the infection risks of COVID-19 at fine spatial scales, e.g., streets and districts, also known as the microscale [7,17]. The urban environment has been noted to considerably influence the spread of COVID-19 [16,17].

According to preliminary results, widely tested microscale urban environmental characteristics have included the urban form, medical and health facilities, residential density, land use, transportation and municipal infrastructures, and green spaces and parks [7,17,18]. Among these, urban density, regarding either population or buildings, has been confirmed to positively affect the spread of infectious diseases, as high-densely urban areas tend to create more social contacts in their daily lives of residents and thus cause higher infection risk [19]. Other than urban density, some public facilities (e.g., clinics) have also been found to increase the infection rate. For example, clinics and restaurants are two built environments that have been shown to influence the number of confirmed cases [15]. This might be due to the high usage of public facilities with increased exposure risk of the virus, and thus confronted with high infection risk [17]. Similarly, road networks seem to increase the risk of COVID-19 transmission in some cities such as Hong Kong, China and Massachusetts, US [15,20]. In addition, some studies have shown that socioeconomic conditions (e.g., race and income) influence infection rates in some American cities [20,21], although these factors have not been significant in other areas, such as Hong Kong and Iran [22,23]. Interestingly, urban climatic conditions (e.g., air temperature and ventilation) are thought to be associated with the pandemic, as warmer temperatures tend to suppress the spread of infectious diseases [24,25]. Moreover, some factors, such as the presence of urban greenspaces, were negatively associated with the infection rate [26].

Although certain studies have highlighted that various built-environment factors influence the infection risk in urban districts, some uncertainties and issues remain. The driving factors have varied across cities and contrasting results have been obtained in some studies. For example, Hamidi [19] indicated that metropolitan size more strongly influenced the transmission risk of COVID-19 than did population density, whereas other researchers have reported the stronger influence of population density [22,27]. In Hong Kong, Yip [15] discovered that social facilities, such as clinics, restaurants, and public markets, are the main drivers influencing the prevalence of COVID-19, while Kwok [22] indicated that urban geometry has a stronger influence on infection rates than do socio-demographic characteristics. Using indicators such as green space, some studies have shown a negative relationship [26], while others have reported a positive association with the spread of COVID-19 [28].

Strong evidence also remains lacking regarding the associations between urban designs and infection risk, which restricts public health officials to simulate at-risk neighbourhoods and urban planners in developing effective health planning strategies to facilitate healthy urban development [19,22]. Specifically, Asian cities are very different from European/American cities because of its high-rise, high-density built environment with a large population density. Without considering factors regarding three-dimensional (3D) urban design/morphology in modelling, it may be difficult to simulate the COVID-19 risk accurately [22].

More importantly, many studies have emphasized the importance of urban morphology to the epidemic; however, most such studies have only examined a few morphological parameters, which becomes a limitation regarding how to consider a range of variables fully reflect the impact of urban morphology on COVID-19 risk in simulation/modelling. For example, Kan [9] observed that a higher building density corresponds to more local confirmed cases, while Guan [8] suggested that road network settings contribute more to the infected cases than other factors. Compared with the two-dimensional (2D) building density, the 3D morphological parameter of building floor ratio, was found to have more significant influence on the infections at Wuhan [29], nevertheless, a contrasting result was obtained at London [18]. Similar with building floor ratio, sky view factor is another typical 3D morphological parameter, which was found to be a good indicator in modelling infection risk [22,28]. Moreover, urban climatic conditions (e.g., urban ventilation) have recorded to be associated with the pandemic, as finer ventilation conditions tend to suppress the spread of infectious diseases [30,31]. As two representative indicators in measuring urban ventilation [32,33], frontal area density and building height to width ratio, might have great potential in modelling infection risk.

To fill this knowledge gap, this study aimed to investigate key attributes of high-rise, high-density built-environment that can be used to develop prediction model of COVID-19 risk. All the above-mentioned urban morphological parameters were tested and systematically assessed in this study, in order to obtain a full understanding about the impact of urban morphology on the transmission of infectious disease for modelling, and thus establish appropriate planning parameters and develop indicators of risk reduction before a new pandemic occurs. Specifically, two typical densely populated Chinese cities with high-rise, high density (Hong Kong and Shanghai) were selected as empirical cases in this study. The modelling influences of 2D and 3D urban morphology factors, and socioeconomic, demographic, and natural parameters on infection rates were explored using both correlation and regression methods. The findings will help (1) public health officials to locate at-risk neighbourhoods with high infectious risk for emergency management and (2) urban planners to formulate appropriate prevention and control strategies and policies to ensure the health of the population.

## 2. Methods

### 2.1. Study Area

**Figure 1:**
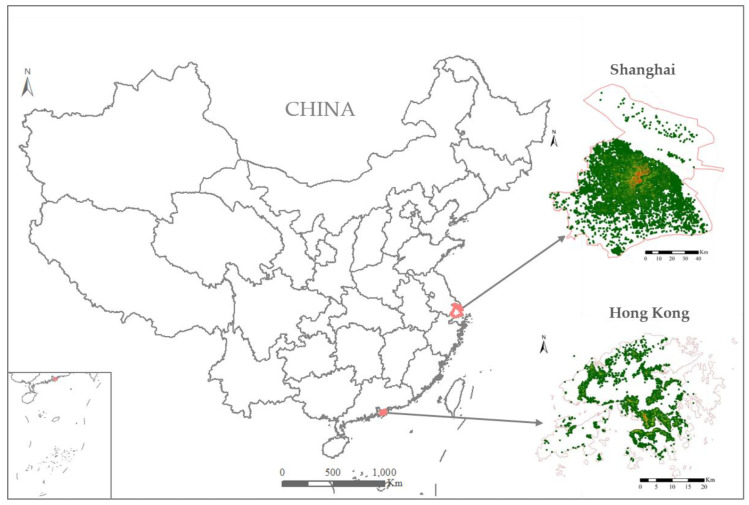
Locations of Hong Kong and Shanghai in China.

Hong Kong (22° N, 114° E) is a Special Administrative Region of China in the eastern Pearl River Delta (Figure 1). It is a high-density city with average and maximum building heights of over 40 m and several hundred meters, respectively. Hong Kong consists of a group of islands and a peninsula, covering an area of 1000 km^2^, with a population of 7.5 million. The city has a hilly topography, and only 25% of the land is developed. Since the initial outbreak, Hong Kong has experienced five waves of COVID-19. The fifth wave emerged in late December 2021 and remains ongoing at the time of drafting this paper. According to the government’s statistical report on Hong Kong, published on 27 September 2022, COVID-19 has infected more than 0.9 million people in Hong Kong and claimed 9921 lives.

Shanghai (31° N, 121° E) is the most prosperous and largest city in China. Similar to Hong Kong, Shanghai is a coastal city, being near the east coast of China (Figure 1). It is located on the alluvial plain of the Yangtze River Delta, with a permanent population of more than 24 million. Shanghai consists of 16 districts, seven of which (Huangpu, Xuhui, Changning, Jing’an, Putuo, Hongkou, and Yangpu) constitute the central urban area. The other regions represent peri-urban or rural areas. Shanghai successfully prevented and controlled COVID-19 transmission until the community outbreak on 1 March 2022, in which 0.6 million people were infected within two months. The city implemented a large-scale lockdown policy and is the third large city in China, following Wuhan and Xi’an, to have been subjected to large-scale and long-term lockdown.

These two cities were selected in this study for the following two reasons. First, both cities are highly dense and compact, as the averaged building height for Hong Kong and Shanghai were 55 m and 17 m, respectively, while the building coverage ratio could reach above 40% at the core areas of both cities. Moreover, both cities faced similar environmental issues, such as poor air quality and ventilation condition [32,34]. Their analyses were thus expected to provide valuable knowledge regarding how to select key attributes for modelling influence of urban density on the spread of COVID-19, which can facilitate the formulation of effective urban planning strategies for the prevention and control of COVID-19 in high-densely urban districts. Second, the cities adopted different prevention and control policies. Shanghai implemented the “dynamic zero COVID-19” policy, which involved mass testing and strict quarantine measures to mitigate the outbreak of COVID-19 before community transmission could occur. The policy adopted in Hong Kong was less stringent, although frequent outbreaks have occurred. As in some Western countries, measures such as vaccination and non-pharmaceutical interventions (e.g., the use of face masks and limits on gathering sizes) have been introduced in both cities. The comparative assessment of both cities with different control policies can help clarify the transmission mechanisms of COVID-19 to facilitate the formulation of adaptable prevention strategies for sustainable development.

### 2.2. Datasets

#### 2.2.1. Infection Data

To identify key attributes for modelling the impact of the urban environment on the spread of COVID-19, data regarding confirmed cases from Hong Kong and Shanghai were collected. Data for Hong Kong, including records of 59,233 cases between 12 February 2020, and 6 February 2022, were collected from its government website (https://data.gov.hk, accessed on 18 March 2022). Each record included the characteristics of the individuals, including age, gender, and location information. Data for Shanghai, including records of 112,956 confirmed cases, were acquired from the official website of the Shanghai municipal health commission (https://wsjkw.sh.gov.cn/, accessed on 15 July 2022). Each record included location information.

**Figure 2:**
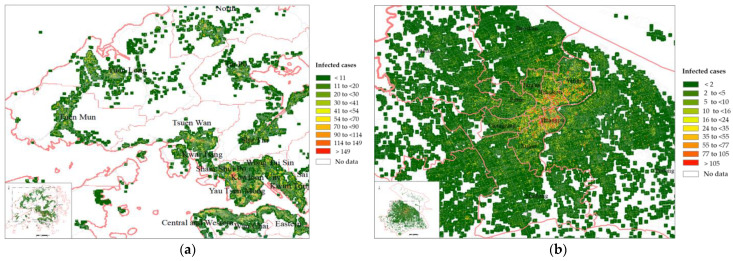
Distribution of confirmed COVID-19 cases in (**a**) Hong Kong and (**b**) Shanghai.

Figure 2a,b shows the distributions of confirmed cases in Hong Kong and Shanghai, respectively. The number of infected cases is presented by a colour gradient, with red and green representing areas with more and fewer infected cases, respectively. High-density and populous urban areas had more infection cases than other regions. Specifically, most of the high-risk areas (marked in red) in Hong Kong were downtown regions such as the Kowloon Peninsula and Central District. Similarly, in Shanghai, the central urban areas (e.g., Huangpu and Hongkou) included more red regions than the other areas (e.g., Baoshan).

#### 2.2.2. Built-Environment Factors

In this study, 13 indicators were used to quantify the high-rise, high-density built environment. These include six urban morphological indicators (sky view factor (SVF); floor area ratio (FAR); frontal area density (FAD); height to width ratio (HW); building coverage ratio (BCR); road density (RD)), four land cover attributes (densities of restaurants (D_Res), bus stations (D_Bus), clinics (D_Cli), and shops (D_Sho)), one environmental index(vegetation index (VI)), and two socioeconomic indices (nightlight intensity (NL); and population (POP)). The selection of all variables was aimed to model COVID-19 risk based on the impacts of 2D/3D urban morphology [22,29], land use facilities, and socioeconomic status [15,22], which have been noted as factors associated with infectious diseases based on prior research [16,17]. The descriptions and basic statistics of these factors are provided in Table 1.

Urban GIS data of the buildings and roads for Hong Kong and Shanghai were collected from the respective planning departments and used to calculate the urban morphological parameters. Data regarding urban facilities, such as restaurants, clinics, and shops were acquired by crawling the point-of-interest data from Baidu maps. The VI was determined using Landsat-8 satellite data. Data regarding human factors (i.e., economic and demographic data) were obtained from the open data products of several organizations. Specifically, the Luojia nightlight data product was used to obtain the economic level, and the global population data product provided by WorldPop (https://www.worldpop.org/, accessed on 19 March 2022) was used to obtain the POP for both cities.

**Figure 3:**
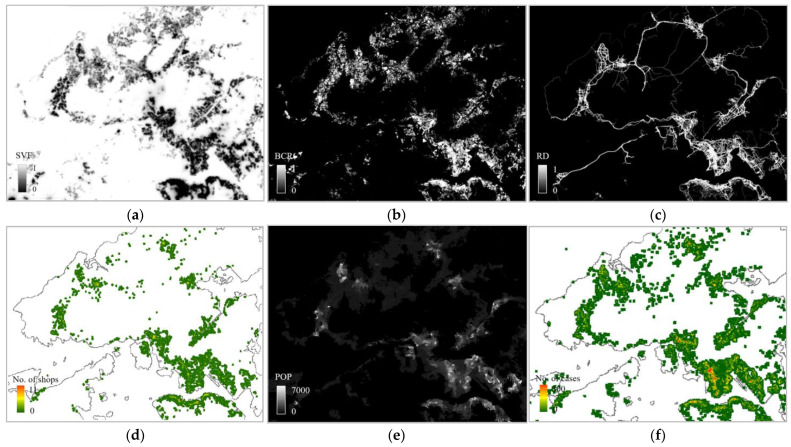
Sample data for Hong Kong: (**a**) Sky view factor; (**b**) building coverage ratio; (**c**) density of roads; (**d**) density of shops; (**e**) population density; (**f**) number of infection cases as a measure of the infection rate.

**Figure 4:**
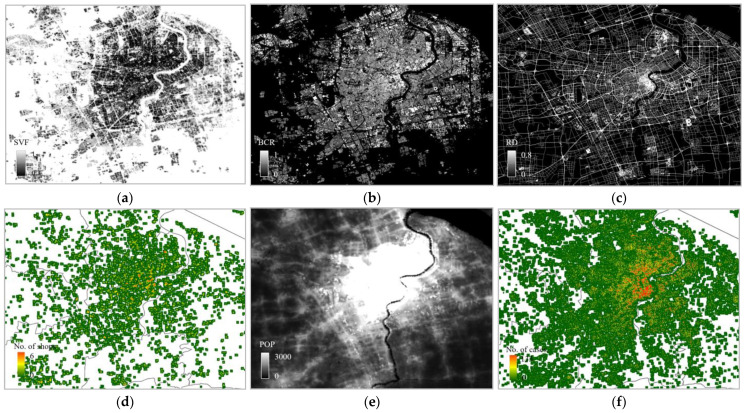
Sample data for Shanghai: (**a**) Sky view factor; (**b**) building coverage ratio; (**c**) density of roads; (**d**) density of shops; (**e**) population density; (**f**) number of infection cases as a measure of infection rate.

To ensure consistency across the factors, all the datasets were spatially aggregated into averaged values based on a defined fishing grid at certain resolutions (e.g., 500 m by 500 m). Figure 3 and Figure 4 show the gridded sample data for both Hong Kong and Shanghai, including the SVF, BCR, RD, D_Sho, POP density, and infections, respectively. The data are consistent in terms of the locations and resolutions.

### 2.3. Identification of Key Attibutes for Modelling COVID-19 Risk

Correlation and regression analysis methods were used to identify key attributes of the built environment that can be used to model the influence of COVID-19 risks. First, a correlation analysis was performed to identify the factors associated with infections. Given that some of the tested variables might not be linearly correlated, the nonparametric correlation methods, including both Spearman and Kendall’s tau-b, were used. Subsequently, multiple linear regression and stepwise linear regression methods were used to analyse the modelled impact of these factors on the infection risks. Given that the distribution of confirmed cases is skewed, a log-transform was applied to obtain an approximately normal distribution [35]. The log-transformed infection data were then further normalized into the range of 0 to 1 as a measure of the infection rate or infection risk.

The multiple linear regression model can be expressed as follows [29]:

y = β_0 + β_1 x_1 + β_2 x_2 +⋯+ β_i x_i + ε(1)

where β_i represents the regression coefficient of the i-th factor, x_i represents the built-environment factor, and y represents the density of infections in a site area.

Based on the above linear regression model, a stepwise regression can be built via a step-by-step construction of a linear regression model. In each step, the method must examine the statistical significance of each independent variable, so that the potential explanatory variable can be added and removed in each iteration. Based on different selection criteria, the stepwise regression model includes different running strategies, such as forward selection, backward elimination and bidirectional elimination. In this study, a bidirectional elimination strategy was used to select most of the appropriate variables.

## 3. Results

### 3.1. Association Analysis for Variable Selection

By dividing each city into multiple grid cells (i.e., 500 m by 500 m), hundreds of valid values were obtained for each variable (i.e., the built-environment factors and infection rate). Using the multiple values of each variable, both Kendall’s tau-b and Spearman correlation analyses were performed to investigate the associations between the urban environment factors and infection rate for modelling variable’s selection.

Table 2 shows the correlation statistics among the 13 tested built-environment factors and the infection rate for Hong Kong and Shanghai. Other than the indicator of HW, all the factors were consistently correlated with the infection rate. As expected, the sky view factor and green space were negatively correlated with the infection rate. Notably, D_Cli, and POP were strongly correlated with the infections in both cities, as the coefficients with either Spearman or Kendall’s tau-b methods were above 0.4. HW and NL were weakly correlated with the infection rate. In particular, HW was not significantly correlated with the infection rate in Hong Kong. Given that HW was weakly correlated with the infection rate in Shanghai; however, it was retained in the following analysis. This result indicated that the infection rate is likely to be driven by a few specific factors rather than all of the variables. According to the correlation analysis, some common factors, such as BCR, D_Cli and POP, have consistent correlation coefficients for both Hong Kong and Shanghai, which indicate that both cities have some similar characteristics in terms of COVID-19 transmission.

### 3.2. Regression for Model Construction

To further identify the factors that were most strongly correlated with the infection rate that can be used to simulate COVID-19 risk, a stepwise regression method was applied to model the infection risk in both cities.

According to the regression results, RD, D_Sho, D_Cli, and POP were the main driving factors for Hong Kong, whereas BCR, RD, D_Sho, D_Cli, and POP were the main driving factors for Shanghai. Table 3 presents the regression results for both cities. The left and right sides show the regression results for Hong Kong and Shanghai, respectively, with the columns presenting the unstandardized coefficients, standardized coefficients, and *p*-values. The R squares of the regression models for both Hong Kong and Shanghai reached 0.60 and 0.49, respectively, indicating that most of the variability in the infections for both cities could be explained.

### 3.3. COVID-19 Risk Mapping

**Figure 5:**
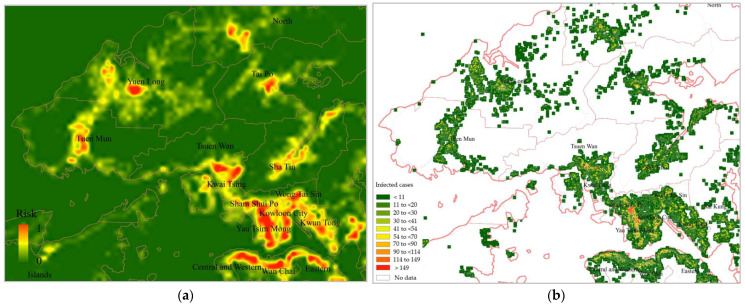
Mapped infection risk in Hong Kong using the proposed regression model. (**a**) Predicted infection risk map; (**b**) Actual observed infected cases as reference.

**Figure 6:**
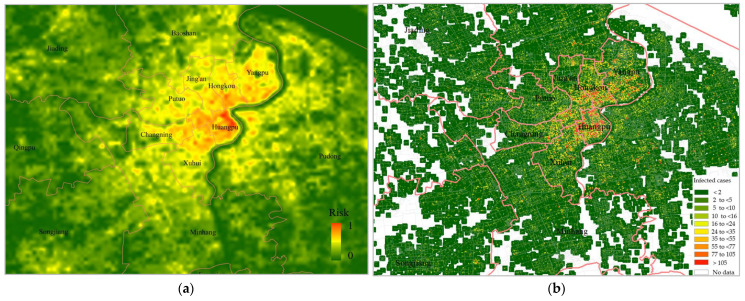
Mapped infection risk in Shanghai using the proposed regression model. (**a**) Predicted infection risk map; (**b**) Actual observed infected cases as reference.

The stepwise models from the above results were used to simulate infection risk maps for Hong Kong and Shanghai, as shown in Figure 5a and Figure 6a, respectively. Red and green indicate areas with high and low infection risks, respectively. The observed infection cases in both cities are presented in Figure 5b and Figure 6b for comparison. Compared with the actual observations, the predicted results provide finer spatial detail regarding the high-risk areas and can clarify the infection risk for regions in which actual infection data are not available.

Based on these results, it is apparent that most of the high-risk neighbourhoods (areas with a red colour) are located in the core urban areas of both cities, such as Yau Tsim Mong and Wan Chai districts, Hong Kong, and Huangpu district, Shanghai. These results might be vital in assisting urban planners to develop reasonable prevention and control plans in advance.

## 4. Discussion

This study identified key attributes of built environment and developed empirical models that can be used to simulate COVID-19 risk in high-rise, high-density cities based on case studies from two Chinese cities with similar urban morphology. The regression results for both cities highlighted that several urban features, such as the BCR, RD, POP, D_Sho, and D_Cli, considerably useful to modelling community transmission risk of COVID-19. All these important parameters could be classified into three categories, including urban morphological attributes (e.g., BCR and RD), socio-economic activities (e.g., shops and clinics), and demographic characteristic at the neighbourhoods.

The variable selections of our empirical models are consistent with results of previous studies. Take the impact of building density for example, Kan [9] and Hamidi [19] confirmed that the urban density took effect for some US cities and Hong Kong, nevertheless, a contrasting result was recorded at London [18], which might be due to the impact of some control variables (i.e., socioeconomic factors). Other than building density, dense road network was found to contribute more infections in high-densely cites of Hong Kong and Shanghai, whereas similar discovers were only found for some cities with low-rise low-dense urban morphology [8]. Results of this study also indicated that POP takes an important effect at the communities, as the overcrowding increases the opportunities with face-to-face infections. Similar findings have been recorded in previous studies [22,27]. Moreover, this study indicated that social activities have made almost equal contributions to the prevalence of COVID-19, compared with urban morphology as well as POP, as the standard regression coefficients of different variables were comparable for both cities (see Table 3). Similar findings were also obtained in Hong Kong by Kwok [22]. Overall, our empirical models were accurate, and this approach of model development could be applied to other cities with similar urban environment.

However, as it is a study for model development, uncertainty regarding this empirical model should be noted. Thus, the later subsections included (1) to describe the performance and spatial uncertainty of potential variables for future modelling, and (2) to identify key messages for planning recommendations and public health management that can be extracted from our results.

### 4.1. Performance of Different Factors

**Figure 7:**
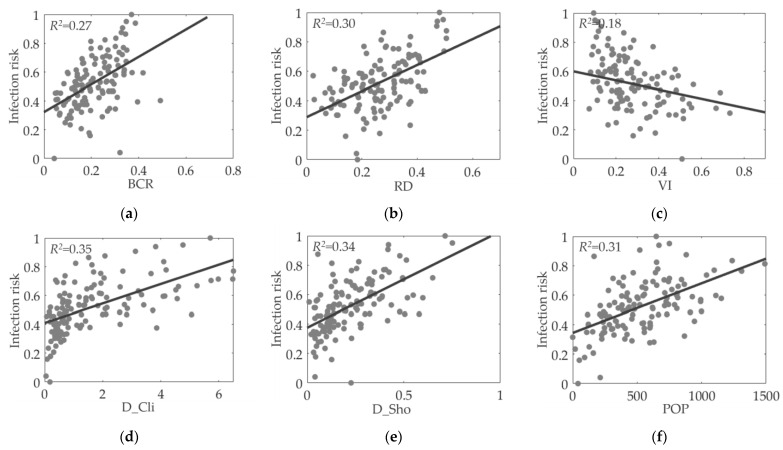
Impact of urban environment features on COVID-19 infection rate in Hong Kong: (**a**) Building coverage ratio; (**b**) Road density; (**c**) Vegetation index; (**d**) Density of clinics; (**e**) Density of shops; (**f**) Population.

**Figure 8:**
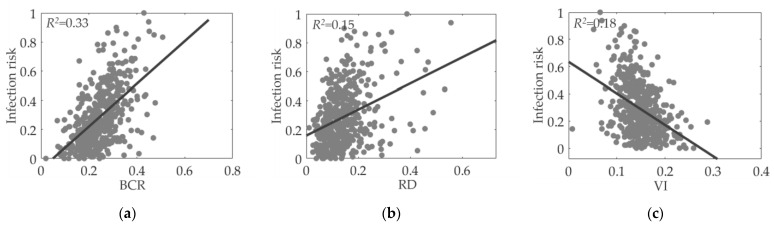

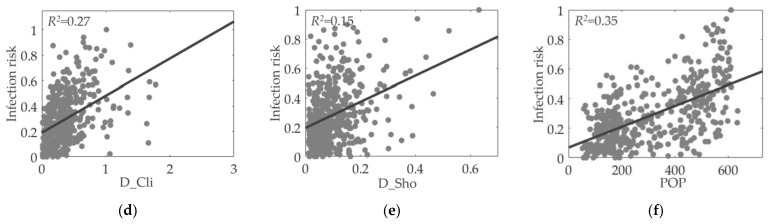
Impact of urban environment features on COVID-19 infection rate in Shanghai: (**a**) Building coverage ratio; (**b**) Road density; (**c**) Vegetation index; (**d**) Density of clinics; (**e**) Density of shops; (**f**) Population.

According to the correlation and regression results, certain factors, such as BCR, RD, POP, and D_Sho, were strongly correlated with the infection rate. Scatterplots with fitted lines were constructed to investigate the influence of several typical factors (BCR, RD, POP, D_Sho, D_Cli, and VI) on the infection rate. Figure 7 and Figure 8 show these scatterplots between the selected factors and the infection rate in Hong Kong and Shanghai, respectively. The x and y coordinates represent the factor and the normalized infected cases, respectively. The R squared value for each factor is also provided to evaluate how closely the points are fitted to the trendline.

The following conclusions were made. First, most of the selected factors were linearly correlated with the normalized infection rate, although the fitted lines of some indices are not that satisfactory, such as the VI for both cities. Second, the impacts of different factors varied across cities. For example, in Shanghai, BCR and POP (R squared values of more than 0.3) were more strongly correlated with the infection rate than were other indices (R values below 0.3). In contrast, the dominant factors for Hong Kong were D_Cli and D_Sho. Third, no single factor could explain the infection rate in communities. The consideration of multiple factors, particularly the combination of both the built environment and socioeconomic factors, enhanced the prediction and simulation of infection risks for both cities.

### 4.2. Scale Effect

To clarify the driving force associated with all factors at different scales, the impact of all variables on infection risk at different scales was analysed. To conduct a valid statistical analysis, the spatial scale could not be excessively large, as the sample size may be insufficient. Moreover, the statistical unit could not be too large owing to the limited data on confirmed cases in both cities. Thus, spatial scales of 200 m, 400 m, and 600 m were selected for Hong Kong, while 300 m, 600 m, and 900 m were selected for Shanghai.

Table 4 presents the stepwise regression results at different spatial scales; based on this information, the following conclusions can be derived.

First, the influencing factors varied across scales. The effect of many factors was notable at finer spatial scales, whereas few factors influenced the infection rate at larger scales. For example, in Hong Kong, seven factors influenced the infection rate at the scale of 200 m, while fewer factors were influential as the scale increased to 600 m. Second, certain key factors (e.g., BCR and POP) remained dominant at all scales, indicating that these factors might drive the community spread of COVID-19. In addition to BCR and POP, RD was an influential factor at finer spatial scales (e.g., 200 and 400 m). However, its impact disappeared at larger scales for both cities. Third, the results of both cities were similar. Some common factors, including POP and BCR, greatly impacted the community infection rate in both cities. Other than POP and BCR, socioeconomic activities, such as D_Sho and D_Cli, also affected both cities, although D_Sho was more significant in Hong Kong, and D_Cli in Shanghai. This difference might be attributable to the distinct control policies of the two cities: Hong Kong citizens were free to shop outdoors during the pandemic, while most citizens of Shanghai could not, which reduced the risks associated with outdoor shopping activities in the latter city. Fourth, the scale of 400–600 m was suitable for the analysis as it reflected the spatial details at the community level of both cities and ensured an acceptable prediction accuracy. This scale was also recommended by Niu [29].

### 4.3. Implications

Generally, our results showed the importance of developing empirical models for infection risk assessment and mapping. Rapid acquisition of fine-scale epidemic data is crucial for developing epidemic prevention and control policies. The lack of precise infection data (e.g., before the outbreak of an epidemic) limits the assessment of infection risk and formulation of appropriate prevention and control plans, which are important to the urban planning of healthy cities [36]. This study provided evidence that the microscale urban environment is strongly associated with epidemic disease transmission and proposed a rapid and efficient epidemic simulation method.

Considering the impact of different factors based on variable selection, the following recommendations can be provided for the design of urban environments to reduce transmission risks. First, the results revealed that urban morphology, particularly BCR and RD, greatly influenced the infection rates in both cities. The finding is partly consistent with the study conducted by Kwok [22] in Hong Kong, but the authors of that study found that the road network was negatively associated with the COVID-19. This difference might be due to the lower number of infections and larger statistical scale used. As in some prior studies [22,37], the proposed urban morphology parameters, including the BCR and RD, could effectively reflect the level of infection risk and thus might guide (1) public health officials to reduce population exposure and social contact in areas with higher BCR and RD and (2) planners in developing appropriate design strategies to improve ventilation and reduce population density in these risky areas for minimizing the infection risks in communities.

Second, areas with higher population densities had a higher infection risk in both cities. However, population density is impossible to reduce naturally. Thus, health officials and planners should develop plans to improve spatiotemporal mobility of different individuals in order to reduce the density of communities in various time slots, despite overall population density cannot be changed. Given that some morphology parameters (e.g., SVF) were highly associated with population density, the infection risk could also be reduced through volumetric design, in order to maintain total population of space usage at the same time reducing people clustering in areas with poor ventilation.

Third, in addition to the urban morphology and POP, social activities, such as those at shops and clinics, may increase COVID-19 transmission risk, as similarly observed in the results of Yip [15]. Given that these factors reflect the flow of the population, it can be concluded that the transmission risk is considerably influenced by population movement. Thus, the spatial scales of public services must be appropriately designed, and population mobility patterns must be changed to ensure healthy cities. Particularly, whether centralized urban design with huge population density in several central business districts or scattered urban design with multiple small blocks having a high land use mix should be considered.

Fourth, although the performance of negative indicators, such as HW and green space, had some uncertainties, their negative correlations with the infection risk are clear in this study, which might indicate that the transmission risk of COVID-19 could be reduced with finer ventilation conditions and more green space.

## 5. Conclusions

Statistical methods were applied to infection data from two Chinese cities to develop empirical model for assessing impact of microscale urban environment features (e.g., urban morphological indices, green spaces, urban facilities, and socioeconomic and demographic data) on transmission and infection rates. A correlation analysis was performed to identify key urban environmental factors associated with the infection rate for modelling. Moreover, a stepwise regression method was used to evaluate the impacts of different factors and their modelling capabilities.

Experimental results indicated that the results of empirical models for both cities were similar. Some common factors, including urban density, population, and social activities, were noted to influence the spread of COVID-19 in communities for both cities, which were suitable for model development. The difference is that different socioeconomic factors take the main effect, as the density of shops was more significant in Hong Kong, and density of clinics in Shanghai. This difference might be attributable to the distinct control policies of both cities, as Hong Kong citizens were free to shop outdoors during the pandemic, while most citizens of Shanghai could not. Moreover, the impact of the factors varied across scales. Specifically, as the scale increased, the influence of several factors disappeared. Factors such as building density and social activities had an impact on larger scales, while some 3D urban morphological parameters, such as SVF and FAR, only had an impact on smaller scales.

Overall, this study (1) highlighted the notable attributes of the urban environment that can be used to model the transmission mechanism of infectious disease at the community level and (2) clarified the modelled influence of various built-environment factors on the infection rate along with their scaling effects. An efficient infection risk warning model was built and verified. This model can be used to identify high-risk urban areas in advance. The proposed methods and findings can provide a reference for epidemic risk assessment and promote the development of reasonable prevention and control strategies.

## Figures and Tables

**Table 1:** Built-environment factors and their descriptions.

Variable	Median (IQR)	
	Hong Kong	Shanghai
Amount of sky that can be seen from the ground (SVF)	0.54 (0.43–0.65)	0.68 (0.61–0.75)
Ratio of a building’s floor area to the site area (in which the building is located) (FAR)	10.20 (7.52–14.90)	4.65 (3.42–5.82)
Density of a building’s frontal area to the site area (FAD)	0.36 (0.26–0.49)	0.17 (0.11–0.23)
Ratio of the average building height to the width of the street abutting the building (HW)	3.52 (2.58–4.79	1.57 (1.23–1.89)
Ratio of the building coverage area to the site area (BCR)	0.20 (0.14–0.28)	0.24 (0.19–0.29)
Ratio of the road area to the site area (RD)	0.26 (0.19–0.35)	0.13 (0.09–0.17)
Number of restaurants in a site area (D_Res)	1.23 (0.59–2.49)	1.05 (0.53–1.73)
Number of bus stations in a site area (D_Bus)	0.62 (0.37–0.84)	0.72 (0.39–1.40)
Number of clinics in a site area (D_Cli)	1.14 (0.52–2.14)	0.25 (0.12–0.43)
Number of shops in a site area (D_Sho)	0.19 (0.10–0.32)	0.09 (0.05–0.13)
Ratio of the vegetation area to the site area (VI)	0.23 (0.16–0.34)	0.15 (0.13–0.17)
Nightlight intensity in a site area (NL)	72.27 (52.17–87.05)	37.08 (31.66–43.97)
Population of a site area (POP)	502.9 (301.8–724.4)	268.1 (161.6–470.7)

**Table 2:** Correlation coefficients between the selected built-environment features and COVID-19 risks for Hong Kong (HK) and Shanghai (SH).

Variable	Hong Kong		Shanghai	
	Kendall’s Tau-b	Spearman	Kendall’s Tau-b	Spearman
Sky view (SVF)	−0.38 **	−0.54 **	−0.45 **	−0.64 **
Floor area (FAR)	0.30 **	0.42 **	0.42 **	0.59 **
Frontal area (FAD)	0.37 **	0.52 **	0.43 **	0.60 **
Height to width (HW)	0.03	0.05	0.22 **	0.32 **
Building coverage (BCR)	0.37 **	0.53 **	0.49 **	0.67 **
Road (RD)	0.36 **	0.52 **	0.22 **	0.31 **
Restaurant (D_Res)	0.47 **	0.54 **	0.35 **	0.49 **
Bus (D_Bus)	0.36 **	0.52 **	0.36 **	0.51 **
Clinic (D_Cli)	0.48 **	0.65 **	0.43 **	0.60 **
Shop (D_Sho)	0.44 **	0.61 **	0.25 **	0.36 **
Green space (VI)	−0.26 **	−0.37 **	−0.32 **	−0.44 **
Nightlight (NL)	0.14 *	0.23 *	0.22 **	0.31 **
Population (POP)	0.41 **	0.57 **	0.46 **	0.64 **

Note: * *p* value < 0.05, ** *p* value < 0.01.

**Table 3:** Stepwise regression results (e.g., regression coefficients and p values) for Hong Kong and Shanghai.

Factor	Hong Kong			Shanghai		
	Unstandardized	Standardized	*p*-Value	Unstandardized	Standardized	*p*-Value
Constant	0.31		0.00	−0.02		0.43
BCR				0.86	0.27	0.00
RD	0.67	0.29	0.00	0.30	0.10	0.00
D_Cli	0.03	0.18	0.04	0.13	0.18	0.00
D_Sho	0.31	0.20	0.02	0.29	0.09	0.01
POP	0.003	0.40	0.00	0.001	0.30	0.00

Hong Kong: *R*^2^ = 0.60, *F* = 45.35, *p* < 0.01. Shanghai: *R*^2^ = 0.49, *F* = 87.28, *p* < 0.01.

**Table 4:** Stepwise regression results at different spatial scales for Hong Kong and Shanghai.

Variable	Standardized Coefficients					
	Hong Kong			Shanghai		
	200 m × 200 m	400 m × 400 m	600 m × 600 m	300 m × 300 m	600 m × 600 m	900 m × 900 m
SVF	−0.28 **	-	-	−0.56 **	-	-
FAR	−0.18 **	-	-	−0.50 **	-	-
FAD	-	-	-	0.30 **	-	-
HW	-	-	-	−0.16 **	−0.09 *	-
BCR	-	0.21 **	0.30 **		0.30 **	0.23 **
RD	0.18 **	0.26 **	-	0.15 **	0.10 **	-
D_Res	-	-	-	0.10 **	-	-
D_Bus	-	-	-	-	-	-
D_Cli	0.23 **	-	-	0.06 *	0.23 **	0.35 **
D_Sho	0.13 **	0.29 **	0.39 **	-	-	-
VI	-	-	-	−0.08 **	-	-
NL	0.08 *	-	-	-	-	-
POP	0.19 **	0.32 **	0.35 **	0.24 **	0.36 **	0.39 **
	*R*^2^ = 0.31	*R*^2^ = 0.51	*R*^2^ = 0.65	*R*^2^ = 0.43	*R*^2^ = 0.58	*R*^2^ = 0.69

**: *p* value < 0.01; *: *p* value < 0.05.

## Data Availability

Not applicable.
